# Comparison of Causality Network Estimation in the Sensor and Source Space: Simulation and Application on EEG

**DOI:** 10.3389/fnetp.2021.706487

**Published:** 2021-09-29

**Authors:** Christos Koutlis, Vasilios K. Kimiskidis, Dimitris Kugiumtzis

**Affiliations:** ^1^ Information Technologies Institute, Centre of Research and Technology Hellas, Thessaloniki, Greece; ^2^ 1st Department of Neurology, Medical School, Aristotle University of Thessaloniki, Thessaloniki, Greece; ^3^ Division of Electronics and Computing, Department of Electrical and Computer Engineering, Aristotle University of Thessaloniki, Thessaloniki, Greece

**Keywords:** multi-channel EEG analysis, sensor space analysis, source space analysis, brain networks, Granger causality, sLORETA

## Abstract

The usage of methods for the estimation of the true underlying connectivity among the observed variables of a system is increasing, especially in the domain of neuroscience. Granger causality and similar concepts are employed for the estimation of the brain network from electroencephalogram (EEG) data. Also source localization techniques, such as the standardized low resolution electromagnetic tomography (sLORETA), are widely used for obtaining more reliable data in the source space. In this work, connectivity structures are estimated in the sensor and in the source space making use of the sLORETA transformation for simulated and for EEG data with episodes of spontaneous epileptiform discharges (ED). From the comparative simulation study on high-dimensional coupled stochastic and deterministic systems originating in the sensor space, we conclude that the structure of the estimated causality networks differs in the sensor space and in the source space. Moreover, different network types, such as random, small-world and scale-free, can be better discriminated on the basis of the data in the original sensor space than on the transformed data in the source space. Similarly, in EEG epochs containing epileptiform discharges, the discriminative ability of network topological indices was significantly better in the sensor compared to the source level. In conclusion, causality networks constructed at the sensor and source level, for both simulated and empirical data, exhibit significant structural differences. These observations indicate that further studies are warranted in order to clarify the exact relationship between data registered in the sensor and source space.

## 1 Introduction

There is an increasing interest in neuroscience in working on the true current distribution of the sources in the grey matter of the brain, termed source space, and not on the extracranial electroencephalograms (EEG) or magnetic encephalogram (MEG) recordings, termed sensor space (De Vico Fallani et al., 2010; Lehmann et al., 2014). Many strategies have been proposed in order to obtain the primary current distribution of the sources in the brain from EEG or MEG. These are basically head models that take into account the volume conduction of the brain and other properties in order to estimate the activity of the initial sources that explain best the extracranial electric potential measurements. Alternatively, it has also been proposed to reconstruct the electrostatic field within a predefined cubic grid of center points, based on the assumption of an ellipsoid and electromagnetically homogeneous head model, without using dipole modeling or other priors, an approach called 3D vector field tomography (Papadaniil and Hadjileontiadis, 2015; Papadaniil et al., 2016). The methods of source reconstruction are separated in two main classes, the over-determined and the under-determined inverse models (Michel et al., 2004). The over-determined inverse models presuppose that a small number of points in the source space is capable of explaining the extracranial measurements sufficiently. In this case, a unique solution, in terms of source location and current activity, is provided when the number of parameters to be estimated is less or equal to the number of sensor space channels. On the contrary, the under-determined inverse models consider a dense three dimensional (3D) grid of points in the brain having fixed positions and being many more than the sensor space channels, which leads to infinite solutions, as stated first in (Helmholtz, 1853). The objective for these models then is to determine a unique optimal source electric activity distribution over the grid of points.

In recent years, the concepts of Granger causality (Granger, 1969) and causality networks are of increasing interest in many branches of science such as finance (Hong et al., 2009; Billio et al., 2012), socioeconomics (Bollen et al., 2011), climatology (Runge et al., 2015; Dijkstra et al., 2019) and neuroscience (Kugiumtzis and Kimiskidis, 2015; Porta and Faes, 2016; He et al., 2019; Rossini et al., 2019). Intuitively, given two variables, *X*_1_ and *X*_2_, the Granger causality from variable *X*_1_ to variable *X*_2_ exists if the past and present of *X*_1_ provide information that improves forecasts for the future of *X*_2_. The concept of Granger causality is implemented in a number of measures in the time, frequency and phase domain, and extended to account for the presence of other observed variables and thus estimate only direct causal effects. In several studies, linear and nonlinear as well as bivariate and multivariate causality measures are compared, e.g., see the recent comparative studies in (Bakhshayesh et al., 2019; Siggiridou et al., 2019; Sommariva et al., 2019) and references therein, and it turns out that the most appropriate measures are the direct and nonlinear measures, but these are also the harder to be estimated reliably. The dimension reduction, intrinsically taking place in the algorithm of some Granger causality measures, allows for the estimation of direct causal effects in high-dimensional time series, such as multi-channel EEG. We consider a linear and a nonlinear such measure in this study, the restricted conditional Granger causality index (RCGCI) (Siggiridou and Kugiumtzis, 2016) and the partial mutual information from mixed embedding (PMIME) (Kugiumtzis, 2013), respectively, both found to perform well in high-dimensional settings (Koutlis and Kugiumtzis, 2016; Siggiridou et al., 2019).

The direct measures are particularly relevant for the formation of causality networks from multivariate time series because only direct causal effects are estimated. The causality networks are graphs with nodes that represent the observed variables of a system and connections between nodes that are weighted by the values of the selected causality measure. These networks are usually analyzed with simple metrics of network theory in order to estimate important characteristics of their topology (Rubinov and Sporns, 2010; Fornito et al., 2016; Geier and Lehnertz, 2017). In neuroscience, the estimation of causality effects among brain areas using EEG recordings is a widely used approach for the observation of brain reactions to certain stimuli and for the discrimination of normal and abnormal states of brain function (Schelter et al., 2006; Lehnertz et al., 2014; Fornito et al., 2016).

For the computation of the connectivity in the source space, in some cases the cortical activity may be estimated for a limited number of nodes and that makes the computations cost-efficient (De Vico Fallani et al., 2010), while in other cases, the number of nodes is prohibitive for a multivariate analysis of the estimated cortical activity (Milz et al., 2014). In the latter cases, the usual strategy is to discretize the cortex in different regions of interest (ROIs) and to consider as new nodes the centers of these regions and assign to them the average activity of all nodes of this region (Hata et al., 2016; Toppi et al., 2016). The connectivity of the estimated cortical activity is estimated using the measures of Granger causality discussed above [see also (Lei et al., 2015)], as well as linear measures (Schoffelen and Gross, 2009; De Vico Fallani et al., 2010; Lehmann et al., 2014; Milz et al., 2014) and nonlinear measures (Mulert et al., 2011; Pascual-Marqui et al., 2014) of similarity and synchronization, i.e. phase synchronization or correlation in the time and frequency domain. Recent studies found that the choice of the inverse method impacts the brain connectivity analysis, e.g., see source-space coherence analysis on magnetoencephalogram (MEG) (Hincapié et al., 2017).

To the best of our knowledge there have not been any reports for systematic comparison of the brain connectivity estimated in the sensor and source space. These two approaches are tested in simulation examples and discussed in (Brunner et al., 2016; Van de Steen et al., 2019). Furthermore, the effect of noise and source locations on the estimation of connectivity in source space is studied in (Anzolin et al., 2019). It is well accepted that EEG sensors do not capture signals exclusively from areas beneath EEG electrodes and the location and orientation of the sources influence critically the signals registered at the sensor level (Van de Steen et al., 2019). For that very reason, in the present study, we examine the overall connectivity and not distinguish one-to-one relationship between connections of individual anatomic areas in the sensor and source space, as done in the two studies in (Brunner et al., 2016; Van de Steen et al., 2019). Our study differs from the two studies also in that it assumes the simulated systems in the sensor rather than the source space, where the latter is physiologically more appropriate as sensors do not interact with each other but brain sources do. However, the data at hand are the scalp EEG (sensor space) and it is thus reasonable to assume simulation systems for the observed dynamics. Moreover, the study is on the overall structure of causality networks and not causal relationships of distinct sources as in (Anzolin et al., 2019; Van de Steen et al., 2019), and for this to develop a high dimensional system in the source space under realistic assumptions is a hard task. For the comparison of causality networks in sensor and source space it was reported strong correlation of the global functional connectivity between the two domains on real scalp EEG of eyes open and closed (Lai et al., 2018). We extend this study using effective connectivity measures and a different real EEG setting of changing connectivity structure.

**FIGURE 1:**
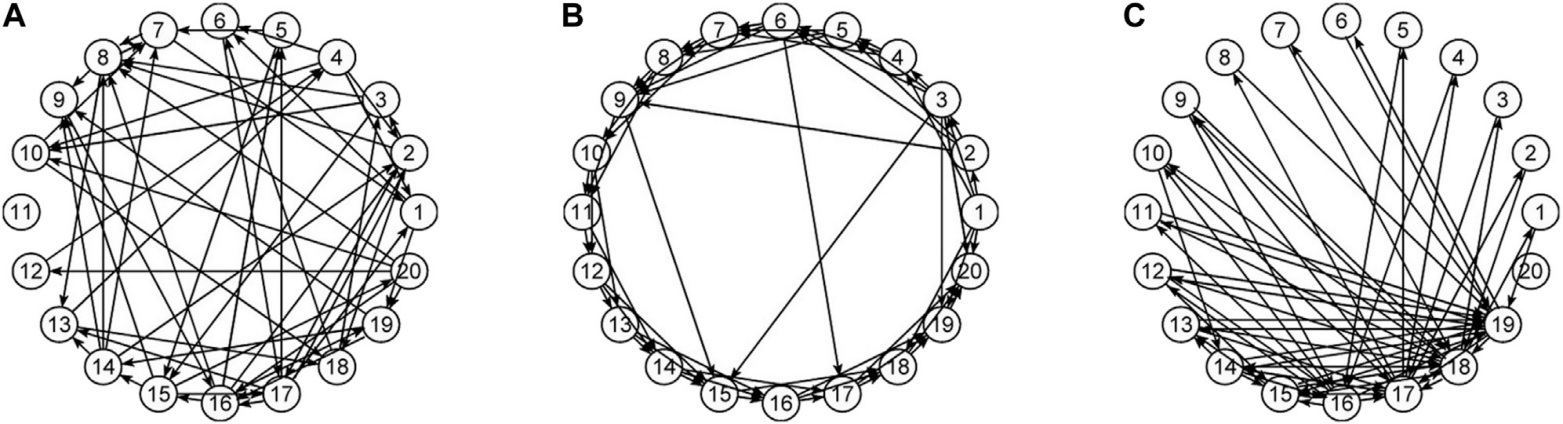
Illustration of network types: **(A)** random, **(B)** small world, and **(C)** scale-free.

In this work, the objective is beyond investigating differences in the estimated connectivity networks in the sensor and source space, which is expected due to the transform and we show it here analytically. Rather, the objective is to apply the same procedures for causality network estimation in the sensor and source space and to compare the causality structures that arise on each of the two workspaces. Though it is not expected to find similar causality structures, an important question is whether the estimated causality structures hold the same discriminative information in any of their characteristics, estimated by network metrics. For this, we performed a simulation study on systems of linear and nonlinear dynamics, having a predefined connectivity structure of one of the three types of random (RAND) (Erdös and Rényi, 1959), scale-free (SCF) (Barabási and Albert, 1999) and small-world (SW) (Watts and Strogatz, 1998) networks. Examples of the network types are presented in Figure 1. The original space of the generated multivariate time series is assumed to be the sensor space and the data are transformed by sLORETA to source space. The objective of the simulation study is the discrimination of different coupling structures (the three network types) by a network characteristic in both spaces. As established in our previous works, the two causality measures RCGCI and PMIME have a very good discrimination capability in the sensor space (the generated time series in the simulation study) and the interest is merely to investigate the deformation of the network structure in the source space. For the neuroscience data analysis, EEG data with episodes of epileptiform discharges (ED) are used in order to compare the causality structure of the brain before, during and after an ED in the sensor and source space.

The paper is organized as follows: in Section 2 the source localization method sLORETA, the causality measures and the network indices are presented, in Section 3 the simulated and the EEG data are briefly discussed, in Section 4 the results are presented and in Section 5 conclusions are given.

## 2 Methods

### 2.1 sLORETA

A commonly used method for the 3D localization of the sources of the electromagnetic activity of the brain is the so-called low resolution electromagnetic tomography, implemented in the LORETA software (Pascual-Marqui et al., 1994). LORETA is suggested as an improvement of the minimum norm estimate (Hämäläinen and Ilmoniemi, 1994). The minimum norm estimate has a main disadvantage in that superficial sources are preferred over deep sources due to the fact that the current density vector is not weighted for the solution of the minimization problem. LORETA overcomes this problem using as weights the norm of the columns of the lead field matrix (the matrix that transforms current density of the sources to extracranial measurements), thus giving truly 3D solutions. Additionally, it provides “smooth” solutions in terms of the minimum weighted Laplacian, which are more plausible as neighboring neurons tend to have coherent waveforms. The way to achieve a meaningful 3D unique solution is by sacrificing spatial resolution, namely the brain volume is discretized in *v* = 2394 voxels considered as separate dipoles and thus the name low resolution electromagnetic tomography. The forward model is defined as:

m=Gd (1)

where $m\in R^{c}$ is the vector of measurements at a certain time point, c∈N is the number of channels, $d\in R^{3v}$ is the vector of current densities of the *v* voxels (for each voxel a 3D vector is considered) and $G\in R^{c\times 3v}$ is the lead field matrix. The discrete problem is:

$${\text{min}}_{d}\|BWd\|\text{, under the constraint }m=Gd$$ (2)

where $W\in R^{3v\times 3v}$ is a diagonal matrix with **W**_*ii*_ = ‖**G**_*i*_‖, **G**_*i*_ is the *i*th column of **G** and **B** is the discrete Laplacian operator 3*v* × 3*v* matrix [for more details on the computation of **B** see the Appendix of (Pascual-Marqui et al., 1994)]. The unique solution for the current densities is:

$$d^{*}=Tm$$ (3)

with **T**=(**WB** ^*T*^ **BW**)^−1^ **G** ^*T*^ [**G**(**WB** ^*T*^ **BW**)^−1^ **G** ^*T*^]^†^, where the † exponent denotes the Moore-Penrose pseudo-inverse.

In (Pascual-Marqui, 2002) an improvement of LORETA is presented suggesting that it achieves zero localization error due to a standardization of the current densities. This improvement is termed standardized low resolution brain electromagnetic tomography (sLORETA) and provides a better resolution with *v* = 6239 voxels. The followed procedure is exactly the same as in LORETA but the estimation d* is finally standardized by its variance. This is actually similar to the work conducted in (Dale et al., 2000) utilizing standardization as well but in a different way from that in sLORETA, which conversely achieves exact localization. More precisely, the covariance matrix $S_{d^{*}}$ of the estimated current density d* is computed and the standardized current densities are:

$${d_{i}^{*}}^{T}{\left\{{\left[S_{d^{*}}\right]}_{ii}\right\}}^{-1}d_{i}^{*}$$ (4)

where $d_{i}^{*}\in R^{3}$ is the current density estimate at the *ith* voxel and ${\left[S_{d^{*}}\right]}_{ii}\in R^{3\times 3}$ is the *ith* diagonal block of matrix $S_{d^{*}}$ .

### 2.2 Causality Network Estimation

For the estimation of the causality networks we use two Granger causality measures, one linear, and one nonlinear, and both in the time domain making use of dimension reduction and being capable of estimating direct causal effects from high-dimensional time series (Koutlis and Kugiumtzis, 2016; Siggiridou et al., 2019).

#### 2.2.1 RCGCI

The measure restricted conditional Granger causality index (RCGCI) is the well-known linear stochastic model-based measure of conditional Granger causality index (CGCI) but based on a restricted (sparse) vector autoregressive (VAR) model (Siggiridou and Kugiumtzis, 2016). The CGCI makes use of the unrestricted VAR model (U-model) of all the lagged variables up to a maximum lag (order) *p* so that in total there are *Kp* terms, where *K* is the number of observed variables. Applying a selection scheme using augmented VAR models, called modified backward-in-time selection, a small subset of lagged variables are selected in the sparse VAR model to constitute the U-model for RCGCI. For high-dimensional systems with sparse coupling structure, the selected subset may have cardinality much smaller than *Kp*. The restricted model (R-model) is derived by excluding the lagged terms of the driving variable *X*, so that if the sparse VAR U-model contains no lagged terms of *X* then RCGCI = 0. If it contains lagged terms of *X* then RCGCI is computed by the log ratio of the fitted error variances of the U-model and R-model, *s*_*U*_^2^ and *s*_*R*_^2^, respectively, as for the CGCI

$${\text{RCGCI}}_{X\to Y}=\log\frac{s_{R}^{2}}{s_{U}^{2}}.$$ (5)

#### 2.2.2 PMIME

The measure partial mutual information from mixed embedding (PMIME) is a normalized version of the partial transfer entropy (Schreiber, 2000; Papana et al., 2012) restricted to the selected mixed embedding vector, formed by selecting progressively from the set of all delayed variables the most relevant ones to the future of the response variable (Kugiumtzis, 2013). The selection relies on information measures, mutual information (MI) and conditional mutual information (CMI). In particular, in the first step of the selection scheme, the lagged variable *w* with the highest mutual information (MI) to the response *Y* (one time step ahead, *y*_*t*+1_), *I*(*y*_*t*+1_; *w*), is selected and constitutes the current subset of lagged variables **w**_*t*_. For each subsequent step, the lagged variable *w* with the highest CMI to the response given the current subset, *I*(*y*_*t*+1_; *w*|**w**_*t*_), is selected and added to **w**_*t*_. The significance of CMI (MI for the first step) is tested at each step and the selection scheme terminates when it is found statistically not significant. Similarly to RCGCI, if the derived subset does not include any lagged terms of the driving variable *X* then PMIME = 0. If it includes, the information of the lagged terms of *X* about the response is evaluated by the CMI of the lagged terms of *X* in **w**_*t*_, $w_{t}^{x}$ , and the response given all other components in **w**_*t*_, normalized by the mutual information of all the lagged terms and the response

$${\text{PMIME}}_{X\to Y}=\frac{I\left(y_{t+1};w_{t}^{x}|w_{t}^{y},w_{t}^{z}\right)}{I\left(y_{t+1};w_{t}\right)},$$ (6)

where $w_{t}^{y}$ and $w_{t}^{z}$ are the lagged terms of the response *Y* and the remaining variables *Z* in **w**_*t*_, respectively, so that $w_{t}=\left[w_{t}^{x},w_{t}^{y},w_{t}^{z}\right]$ .

Both causality measures include an internal criterion to determine the presence of the driving of one variable to another and in that case a positive value of RCGCI or PMIME quantifies the strength of the driving, otherwise the measure is zero. This leads to a weighted matrix with zero and weighted connections of driving strength, thus the binarization of the causality network is performed just by accepting the positive values as existing causal effects.

### 2.3 Network Metrics

We select five network metrics, which capture different characteristics of the network structure (Rubinov and Sporns, 2010). These metrics are computed on the adjacency matrices formed by the positive PMIME or RCGCI values for each ordered pair of variables and regard the causality network estimated from the multivariate time series.

The five network metrics quantify different properties of the network with binary directed or non-directed connections. The centrality property of the network is quantified by the mean, *deg* ^*m*^, and standard deviation (SD), *deg* ^*SD*^, of the degree distribution over the nodes. The characteristic path length, *λ*, is used as index of functional integration. The small-worldness index, *SWi*, quantifies the presence of small-world structure in the network, which suggests the combination of functional segregation and functional integration in a network. As an index of resilience we consider the assortativity coefficient, *r*, quantifying the preference of network nodes to attach to other nodes of similar degree, typically defined as the correlation coefficient between the degrees of two nodes.

### 2.4 Evaluation Index

In the focus of the study is the discrimination of different connectivity structures in the sensor and source space, and we consider the three network types, i.e., random (RAND), small world (SW), and scale-free (SCF) (see Figure 1). We consider a number of realizations of multivariate time series from each network type and compute the causality networks and subsequently the network metrics for each realization. For each pair formed from any two of RAND, SW, and SCF, the evaluation index area under receiver operating characteristic curve (AUROC) (Fawcett, 2006) is employed to quantify the overlapping of the two distributions of the network metric in the two network types. For the simulation study, the AUROC is computed for each pair of network types of the original causality network (given by the system equations) and the estimated causality networks in the sensor and source space.

## 3 Materials

In the simulation study, the synthetic systems are considered to evolve in the sensor space. One could argue to consider the source space instead, but we do not follow this line here for two reasons. First, the assumption of the sensor space as the space of the generated time series is a natural choice since the starting data, the acquired EEG measurements, are in the sensor space. Secondly, the generation of data in the source space is a hard task and can be realized under rather unrealistic simplifications, e.g., assuming the same activity in all vertices (voxels) in a ROI on the cortical mesh (Silva Pereira et al., 2017). We consider three systems of different type. The two first systems are deterministic chaotic dynamical systems while the third is a linear multivariate stochastic process. The two first systems are coupled systems, i.e., are formed from a number of subsystems of the same type coupled to each other. The first system is in discrete time and the second in continuous time.

### 3.1 Simulated Data

The system of coupled Hénon maps (CHM) (Kugiumtzis, 2013) is a system of coupled chaotic maps defined as

$$x_{j,t}=1.4-{\left[\frac{\sum_{i=1}^{K}C_{ij}x_{i,t-1}}{\sum_{i=1}^{K}\Theta \left(C_{ij}\right)}+\left(1-\frac{\sum_{i=1}^{K}C_{ij}}{\sum_{i=1}^{K}\Theta \left(C_{ij}\right)}\right)x_{j,t-1}\right]}^{2}+0.3x_{j,t-2}$$ (7)

where *j* = 1, 2, … , *K* is the variable index, *K* denotes the number of variables and *C*_*ij*_ is the coupling strength (considering *x*_*i*_ as the driving variable and *x*_*j*_ as the response variable). The Θ(*C*_*ij*_) is the Heaviside function, being one if *C*_*ij*_ > 0 and zero if *C*_*ij*_ = 0. For this system, we consider *K* = 20 variables coupled with strength *C*_*ij*_ = *C* = 0.2 for the non-vanishing terms according to each of the three network types (RAND, SW, SCF). For each network type, 50 realizations are generated and the time series have length *N* = 2048.

The system of coupled Mackey-Glass (CMG) (Senthilkumar et al., 2008; Kugiumtzis, 2013) is a system of coupled identical delayed differential equations defined as

$${\dot{x}}_{j}\left(t\right)=-0.1x_{j}\left(t\right)+\overset{K}{\underset{i=1}{\sum }}\frac{C_{ij}x_{i}\left(t-\Delta \right)}{1+x_{i}{\left(t-\Delta \right)}^{10}},$$ (8)

where *j* = 1, 2, … , *K* is the variable index, and Δ is the delay parameter. For this system we consider Δ = 100 time units (for the uncoupled system each equation defines a chaotic system of correlation dimension close to 7) and *C*_*ij*_ = *C* = 0.1. The solution is run in Matlab (Natick, Massachusetts: The MathWorks Inc.) using the function “dde23” and then sampled at 4 time units. Further details for the generation of the CMG time series are given in (Kugiumtzis, 2013). The setting is as for CHM, *K* = 20, 50 realizations of each of the RAND, SW, and SCF network types and *N* = 2048.

The third systems is a linear stochastic VAR process (Basu and Michailidis, 2015) on *K* = 20 variables and order *p* = 1,

$$x_{t}=Ax_{t-1}+e_{t},$$ (9)

where **x**_*t*_ is the state vector of length *K* and **e**_*t*_ is the white noise vector of length *K* following Gaussian distribution with zero mean and unity covariance matrix. The components of the square coefficient matrix *A* are zero or positive determined by the selected network of type RAND, SW, or SCF. Initially, the non-vanishing coefficients are set to 0.9 (the rest are set to zero) and then they are iteratively reduced until the stationarity condition is fulfilled. We get 50 multivariate time series for each of the three network types of length *N* = 2048.

The PMIME is used to estimate the connectivity for the two first nonlinear systems and the RCGCI for the third linear system, as being the most appropriate measures to capture the causality effects in each system. This is so because the objective here is to have an appropriate measure that estimates best the causality network from the time series in the sensor and source space.

**FIGURE 2:**
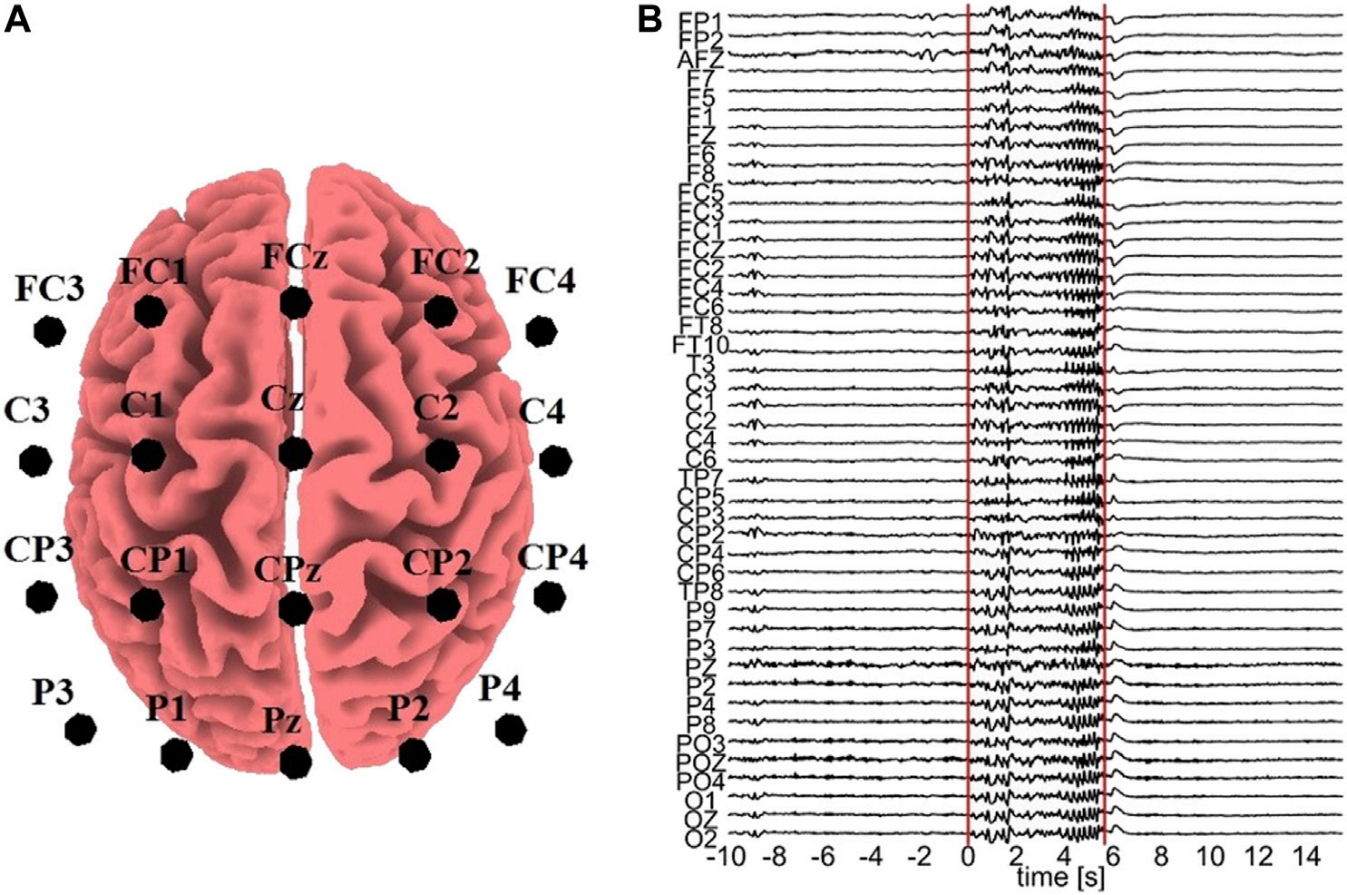
**(A)** Electrode positions assumed for the sLORETA on the simulated data. **(B)** One epoch with an ED episode. The vertical lines represent the start and end points of the ED, respectively, and the time order is with respect to the ED start (0 time at start).

All the computationally generated data are treated as if they were EEG signals (in the sensor space) in order to transform them with sLORETA (to the source space) and the *K* = 20 variables are considered as electrode positions on the scalp, more precisely: FC3, FC1, FCz, FC2, FC4, C3, C1, Cz, C2, C4, CP3, CP1, CPz, CP2, CP4, P3, P1, Pz, P2, P4. These positions are selected in order to cover a sufficient area of the brain rather than concentrating on a small region, as shown in Figure 2A.

For each of the 50 realizations, the hypothesized multi-channel (*K* = 20) time series in the sensor space is transformed with sLORETA to *v* = 6239 source space signals, which consequently are separated in ROIs and averaged, thereby resulting in *K* = 20 source space time series. The ROIs are selected with the option of “all nearest voxels” in the sLORETA software, which separates the voxels in mutually disjoint regions of the nearest to the selected electrodes voxels.

### 3.2 EEG Data

For the real data analysis, EEG data from three epileptic patients at rest are used. In order to avoid the confounding variables of diverse structural substrates and varying localizations of the epileptic zone, which typically characterize focal epilepsies, we used data from patients suffering from Genetic Generalized Epilepsies. From the initial recording of 60 channels, channels containing artifacts were rejected, i.e., 16 channels for subject 1, 9 channels for subject 2, and 14 channels for subject 3. The EEG data of the artifact-free channels were band-pass filtered in (0.01,70) Hz, downsampled to 200 Hz, and re-referenced to infinity (Yao, 2001), a re-referencing scheme found to be more appropriate for connectivity analysis (Qin et al., 2010). Epochs containing epileptiform discharge (ED) were selected, 7 epochs from subject 1, 3 epochs from subject 2 and 10 epochs from subject 3. An exemplary epoch from subject 1 is shown in Figure 2B. Each epoch contains a pre-ED period of 10 s, a spontaneous ED of duration from 2.19 to 5.71 s and a post-ED period of 10 s.

The sLORETA software is used for the transformation of the EEG signals to the source space. First, the electrode positions are selected and then the transformation matrix is generated by the software. Consequently, the signals are transformed to the source space giving a large number of *v* = 6239 time series. In accordance with the simulation study, a number of 44 ROIs corresponding to the 44 scalp electrodes for subject 1 are selected and 44 time series for the source space are obtained, and the same was done accordingly for the artifact-free channels of the other two subjects.

After the transformation of the time series, both the sensor and source time series are split in sliding overlapping windows of 2 s with a sliding step of 0.5 s to profile the brain network characteristics and all their changes during the epochs. The causality estimation is performed with PMIME and then characteristics of the causality networks are estimated by the network metrics previously presented.

## 4 Results

Before presenting the results of the simulation study, it is elaborated that a linear transformation may change the connectivity of a set of variables in a significant degree. Although the employed methodology (sLORETA) is not a linear transformation, the following simplistic example is indicative of the changes a transformation in general can induce. Let us assume a linear VAR(1) process on *K* variables

$$x_{t}=Ax_{t-1}+e_{t},$$ (10)

where the coefficient matrix *A* defines the connectivity matrix of the original system, **x**_*t*_ = [*x*_1,*t*_, *…* , *x*_*K*_,_*t*_] is the vector of the state of *K* variables at indexed time *t* and **e**_*t*_ = [*e*_1,*t*_, *…* , *e*_*K*_,_*t*_] is the vector of noise terms at time *t* having normal distribution *e*_*i*_,_*t*_ ∼ *N*(0, *σ* ^2^), *i* = 1, … , *K*, and covariance matrix $\Sigma_{e_{t}}=\sigma^{2}I_{K}$ , where *I*_*K*_ is the identity matrix of size *K* × *K*. Consider a linear transformation of **x**_*t*_

$$y_{t}=Hx_{t},$$ (11)

where *H* is a matrix of size *K* × *K* with det(*H*) ≠ 0. Solving Eq. (11) with respect to **x**_*t*_ and substituting it in Eq. (10), the VAR (1) for **y**_*t*_ is obtained as:

$$y_{t}=HAH^{-1}y_{t-1}+He_{t}=By_{t-1}+e_{t}^{\prime },$$ (12)

where the input noise vector $e_{t}^{\prime }$ is correlated (the noise covariance matrix is not diagonal) indicating also the presence of instantaneous causality, which however is not relevant here, as we estimate only the lag causality. In analogy to the connectivity of the original VAR(1) system, the connectivity of the transformed VAR(1) system is determined by the coefficient matrix *B* = *HAH* ^−1^. Thus, a variety of different connectivity structures for the transformed system can be obtained, dependent on the form of *H*, with the only constraint being that the initial and the transformed system have connectivity matrices with the same eigenvalues. Also, it is noteworthy that the transformation matrix $H=Q_{A}^{-1}$ , where the columns of *Q*_*A*_ are the eigenvectors of *A*, leads to a diagonal *B* and subsequently a linearly transformed VAR(1) system with no connections among its variables, regardless of its initial connectivity structure.

### 4.1 Simulation Study

**FIGURE 3:**
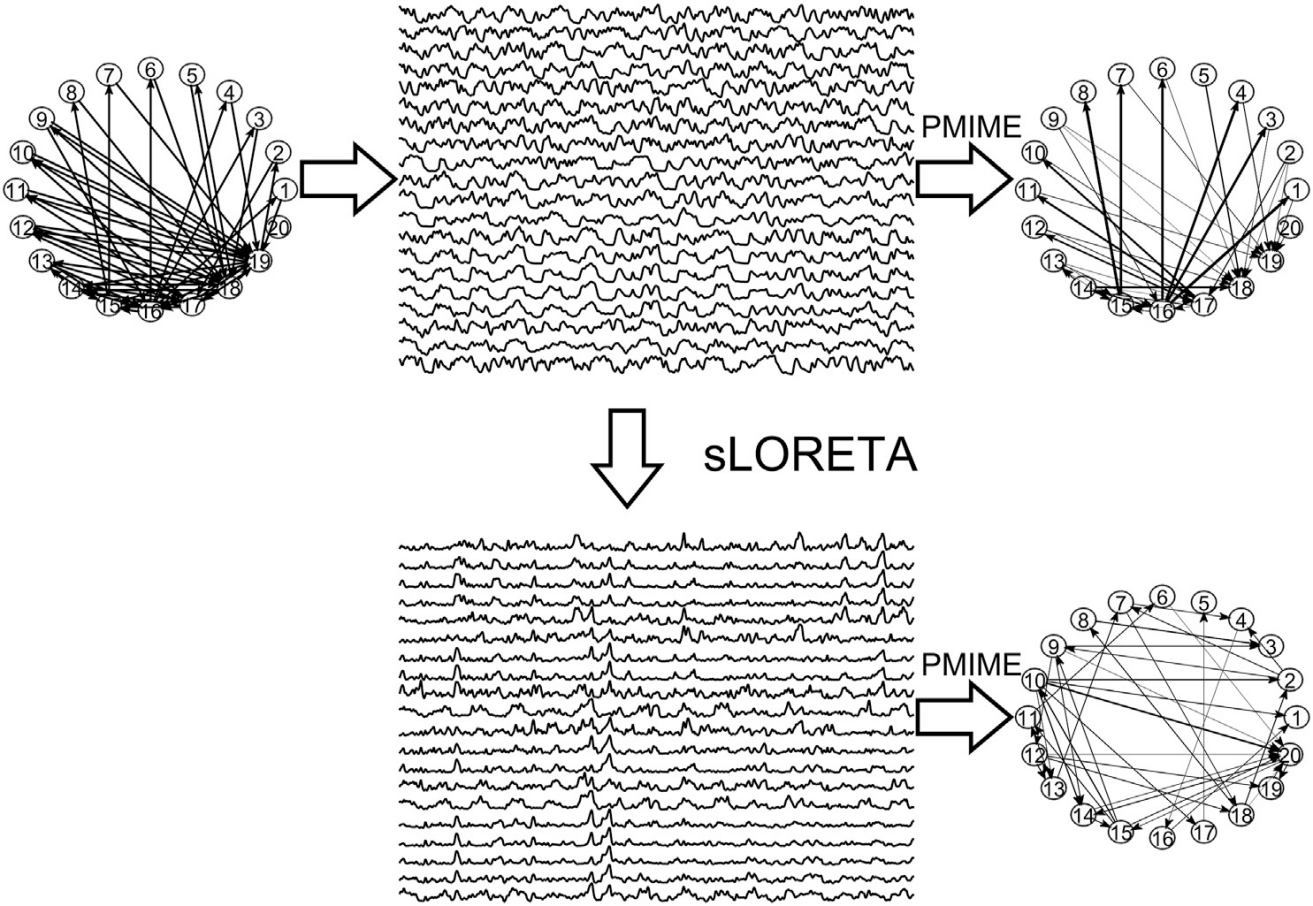
The initial scale-free coupling structure among the variables is used to generate the *K* = 20 time series from the CMG system hypothesized to be in the sensor space. Then, sLORETA derives the respective time series in the source space and finally the PMIME is used to estimate the causal effects and give the causality networks in both the sensor and the source levels.

In Figure 3, the steps of the procedure followed in the simulation study are illustrated in an example for the CMG system. Initially, a network of a predefined type (RAND, SW, or SCF) is defined, in order to form the coupling relationships in the system equations, for the generation of the 50 simulated time series of each network type in the hypothesized sensor space. Then, sLORETA is used to transform the time series to the source space. The causal effects for all pairs of observed variables are estimated by a causality measure (PMIME for CMG in Figure 3) in both the sensor and the source space, resulting in the respective causality networks. It is observed that the form of the time series is rather different before and after the sLORETA transformation. More precisely, the source space time series are less oscillatory and show spikes at various times contrary to the initial time series.

**FIGURE 4:**
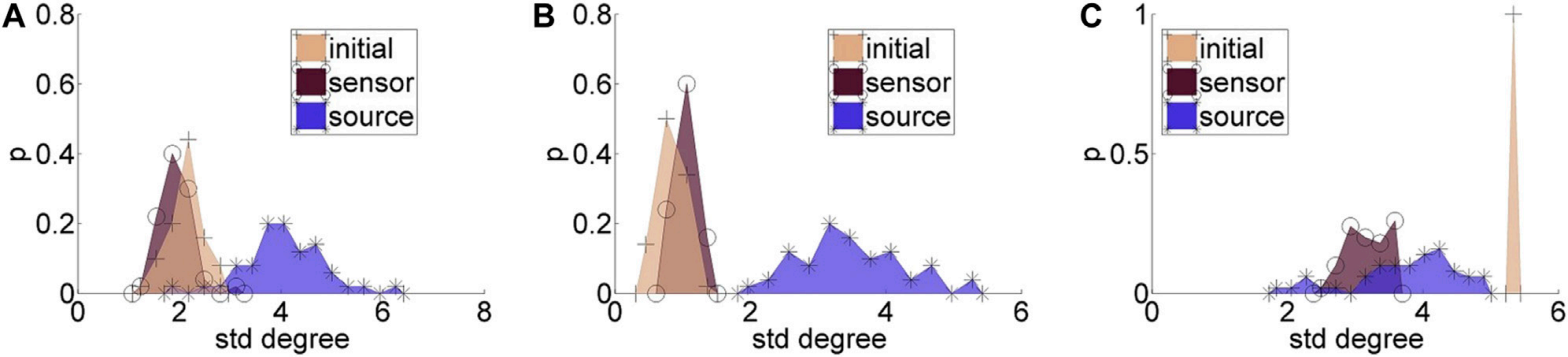
Histograms for the standard deviation of the degree, *deg* ^*SD*^, for 50 realizations of each network type: **(A)** random (RAND), **(B)** small-world (SW), and **(C)** scale-free (SCF). At each panel, there are three histograms, for the initial network, the network estimated in the sensor space, and the network estimated in the source space, as given in the legend.

Regarding the causality network estimation, for the example in Figure 3 the PMIME has a satisfactory performance on the sensor space time series capturing the original scale-free structure. The causality network of the transformed time series in the source space is qualitatively different from the original network, as expected from the previous analytical example. This is quantitatively confirmed for instance by the network metric *deg* ^*SD*^ that takes the values 5.33 and 2.35, respectively, for the initial structure and the source space estimation. In Figure 4, aggregate results from the 50 realizations are shown for all three network structures, i.e., the initial network, the network estimated in the sensor space and the network estimated in the source space. The similarity of the *deg* ^*SD*^ between the original network and the estimated network in the sensor space as well as their dissimilarity to the estimated network in the source space are striking for the cases of RAND and SW networks. For SCF networks, the PMIME does not estimate with the same high accuracy the initial network (see specific results in (Koutlis and Kugiumtzis, 2016)) and therefore the histograms of *deg* ^*SD*^ for the sensor and source space networks differ significantly from the respective histogram for the initial network.

**FIGURE 5:**
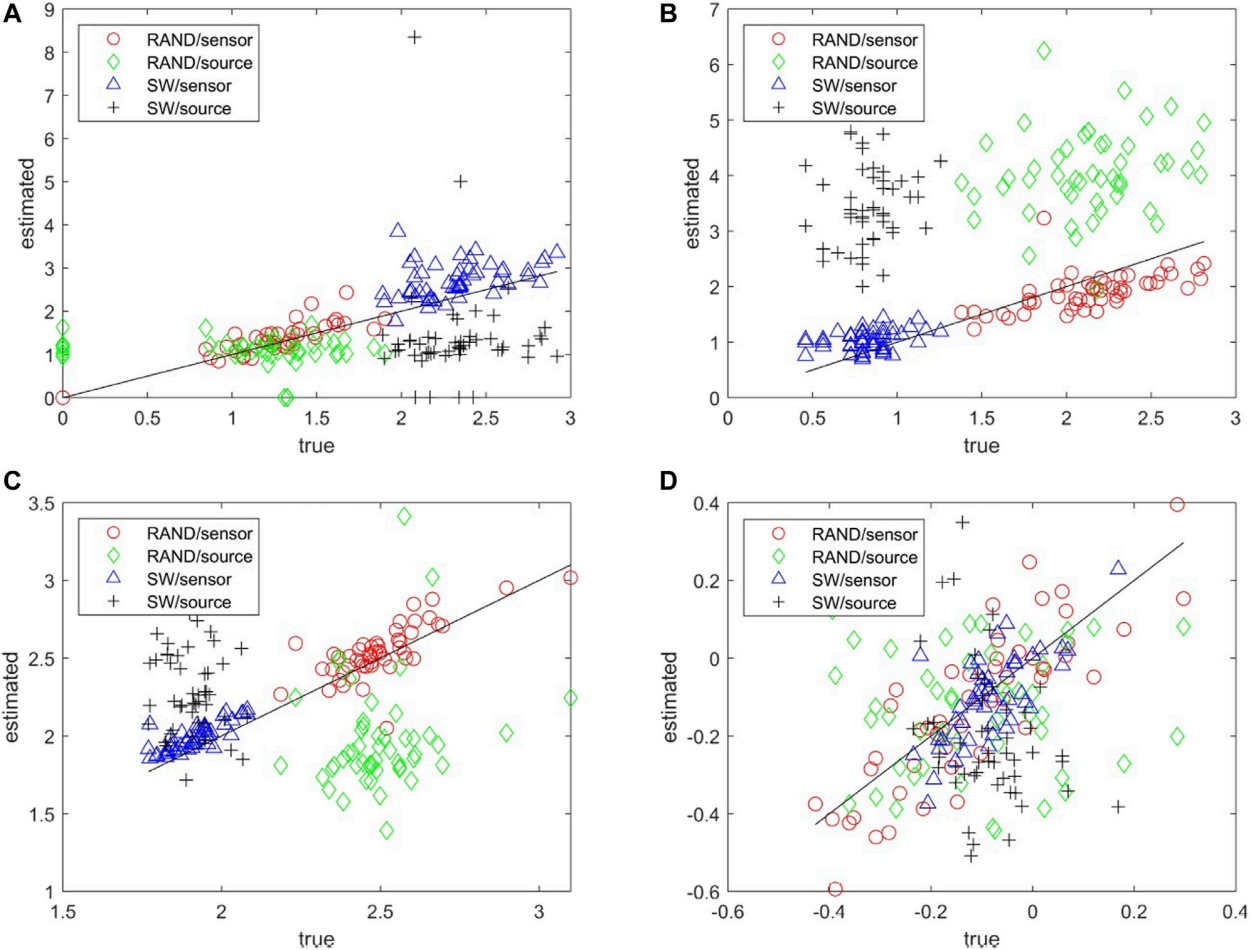
Scatter plots of network metrics estimated on 50 realizations of random (RAND) and small-world (SW) network types, where in the *x*-axis is the metric for the true network and in the *y*-axis for the estimated network in the sensor and source space, as shown in the legend. The metrics are *SWi* in **(A)**, *deg* ^*SD*^ in **(B)**, *λ* in **(C)** and *r* in **(D)**.

The matching of the metrics of the estimated network to the true network can be seen collectively in the scatter plots for each network metric and for both RAND and SW network types in Figure 5. Results are not shown for the characteristic *deg* ^*m*^ being constant for the true networks and the SCF network type as for all but the network metric *r* the metric of the true network is rather constant across the 50 realizations. The network metrics *SWi*, *deg* ^*SD*^, and *λ* computed on the sensor space tend to match well with the respective metrics of the true networks whereas for the source space the respective metrics are more spread and have the tendency to overestimate or underestimate the metrics of the true network (over or under the diagonal in Figures 5A–C, respectively). For the metric *r* in Figure 5D the values of the true networks are at the same range for RAND and SW network types so that at first look *r* values seem to spread about the same for both sensor and source space. A careful look would discern the alignment of the *r* values for the sensor space along the diagonal and indeed the Pearson correlation coefficient values are for RAND network type 0.84 for sensor space as opposed to 0.01 for source space and for SW network type 0.67 and −0.28, respectively.

**FIGURE 6:**
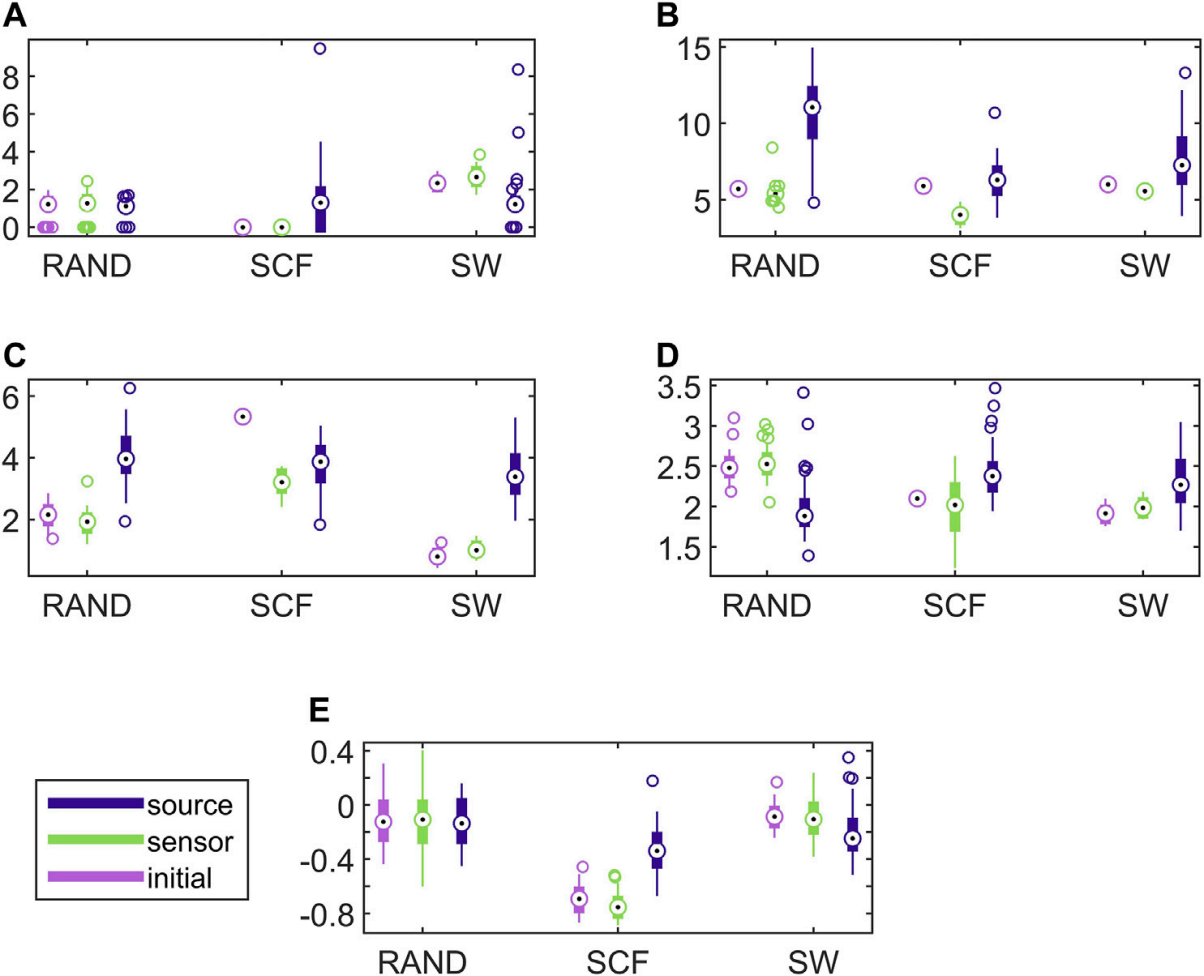
Distribution of network metric values for three different network types (RAND, SW, and SCF) computed on the initial network and on the networks estimated by PMIME on the sensor space and on the source space, as shown in the legend. The PMIME is computed on simulated time series of the CMG system. The metrics are *SWi* in **(A)**, *deg* ^*m*^ in **(B)**, *deg* ^*SD*^ in **(C)**, *λ* in **(D)** and *r* in **(E)**.

Establishing that the connectivity changes significantly under the sLORETA transform, we investigate now whether the transform preserves the discriminative information that allows for correct classification of the different initial coupling structures. For this, we examine the discrimination of the three network types in the sensor and source space. In Figure 6, results for the discriminative ability of the network metrics are presented for the CMG system, where the estimated networks are derived by the PMIME.

It is observed that the values of all network metrics for the initial network and the network estimated on the sensor space range in a similar scale. This includes also the case of *deg* ^*SD*^, where though the initial connections are not preserved well in the sensor space with the PMIME for SCF networks (see Figure 4C), still the SCF networks can be clearly discriminated with *deg* ^*SD*^ from RAND and SW networks. The values of the network metrics for the source space are in some cases similar with those of the initial networks, e.g., *SWi* on RAND network, but in most cases they range in different scales. This result indicates that the transformation to the source space changes significantly the structure of the causality network. The discriminative information of the network metrics, regarding the three network types, remains in the sensor space estimation at a larger degree than in the source space. This result is confirmed by the average AUROC values for the differences in the three pairs of the RAND, SW, and SCF network types, presented in Table 1. While the AUROC values for the networks estimated in the sensor space are high and similar to these for the initial networks, the respective AUROC values for networks estimated in the source space are much lower. However, in almost all of the discrimination tasks of Table 1 we obtain a *p*-value < 0.01 after ANOVA hypothesis testing for equal mean AUROC in the RAND, SW and SCF network types, with the exception of source space estimation of *SWi* on CMG, source space estimation of *SWi* on CHM, source space estimation of *r* on CHM and source space estimation of *r* on VAR.

**TABLE 1:** Average AUROC values from the three AUROC values computed in the three binary classification tasks, RAND vs. SW, RAND vs. SCF, and SW vs. SCF, for the five network metrics (labels in the first row), the three dynamical systems (labels in the first column) and for the initial networks, the networks from the sensor space and the networks from the source space.

		*SWi*	*deg* ^*m*^	*deg* ^*SD*^	*λ*	*r*
CMG	initial	0.999	1	1	0.995	0.856
	sensor	0.997	0.878	0.995	0.828	0.842
	source	0.546	0.839	0.633	0.768	0.713
CHM	initial	1	1	1	0.993	0.866
	sensor	0.99	0.957	0.988	0.924	0.713
	source	0.523	0.781	0.706	0.678	0.613
VAR	initial	0.999	1	1	0.993	0.888
	sensor	0.951	0.953	0.993	0.949	0.729
	source	0.739	0.841	0.855	0.851	0.559

**FIGURE 7:**
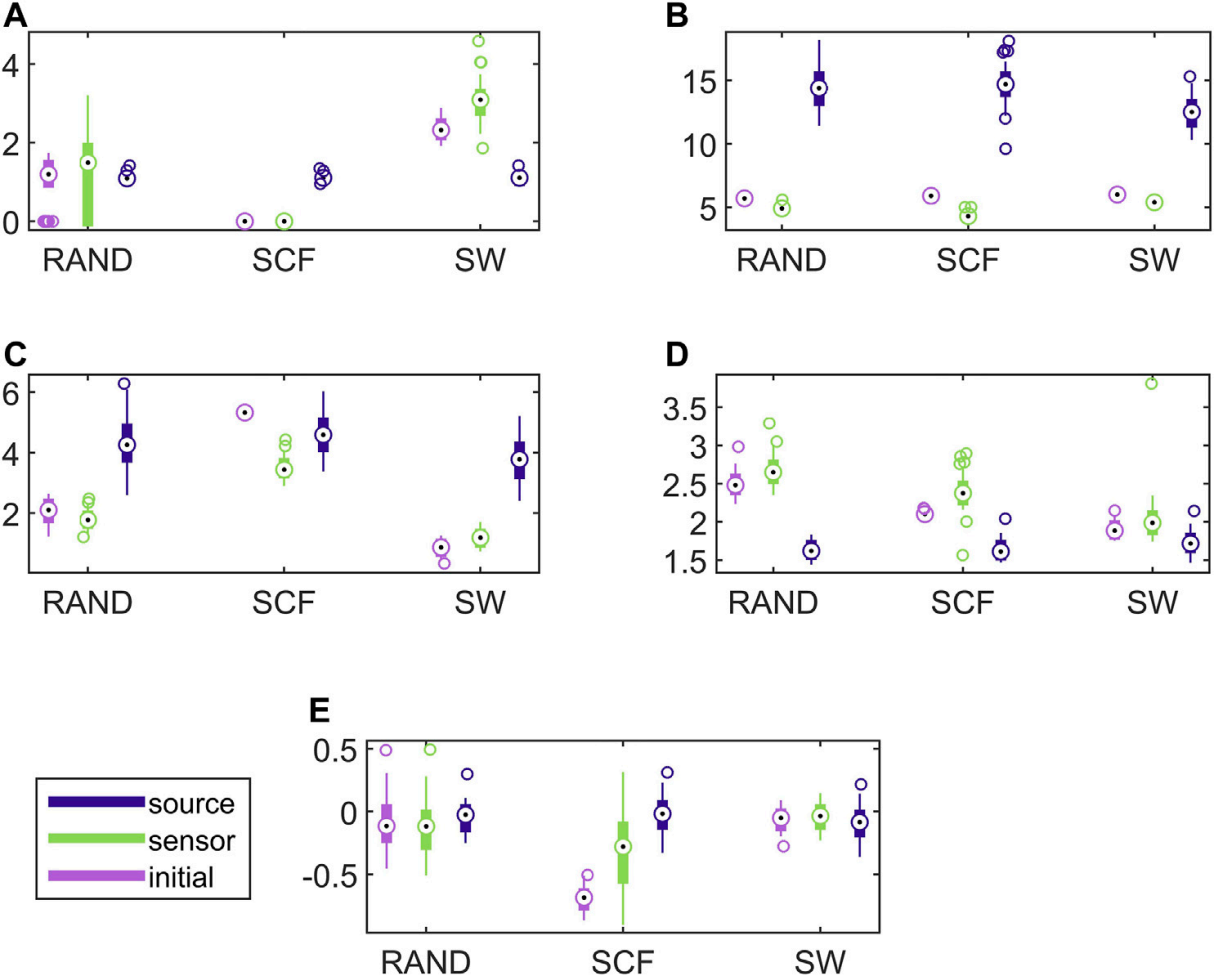
Distribution of network metric values for three different network types (RAND, SW, and SCF) computed on the initial network and on the networks estimated by PMIME on the sensor space and on the source space, as shown in the legend. The PMIME is computed on simulated time series of the CHM system. The metrics are *SWi* in **(A)**, *deg* ^*m*^ in **(B)**, *deg* ^*SD*^ in **(C)**, *λ* in **(D)** and *r* in **(E)**.

In Figure 7, results for the discriminative ability of the network indices are presented for the CHM system in the same way as shown earlier for the CMG system in Figure 6. Similarly to the CMG case, the values of all network indices for the initial network and the network estimated on the sensor space range in a similar scale. In contrast, the values of the network indices for the networks estimated on the source space range in different scales with those of the initial networks in most of the cases. Again, this is confirmed with the results of AUROC in Table 1.

**FIGURE 8:**
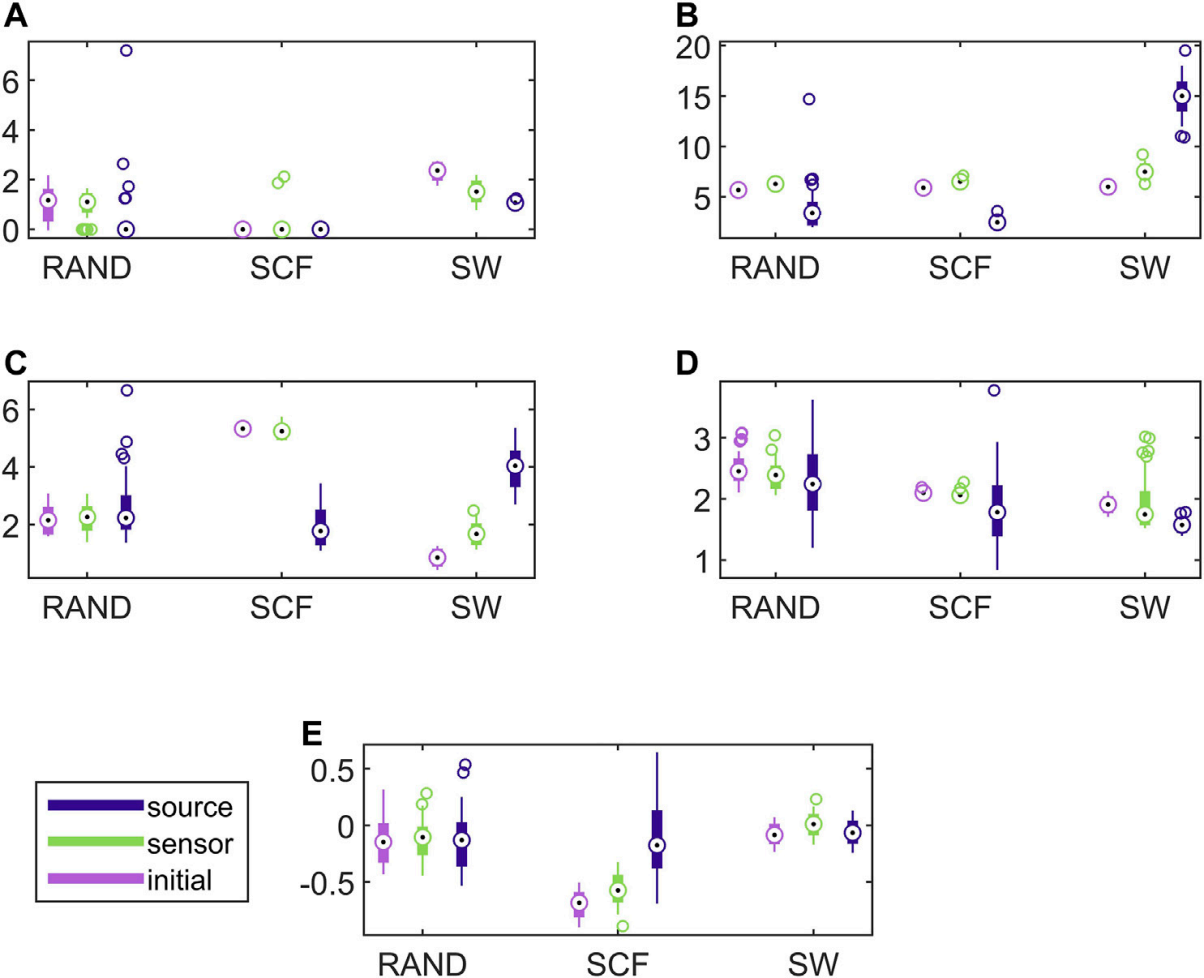
Distribution of network metric values for three different network types (RAND, SW, and SCF) computed on the initial network and on the networks estimated by PMIME on the sensor space and on the source space, as shown in the legend. The PMIME is computed on simulated time series of the VAR system. The metrics are *SWi* in **(A)**, *deg* ^*m*^ in **(B)**, *deg* ^*SD*^ in **(C)**, *λ* in **(D)** and *r* in **(E)**.

In Figure 8, the respective results for the VAR system are presented. Here, the causality networks are estimated with the linear causality measure RCGCI, which is more appropriate (and simple) for the linear VAR process. Regarding the range of network index values, in many cases the initial network, the sensor space network and the source space network share the same range of values, e.g., for the random network type and all measures or the scale-free network and *SWi* and *λ*. Also, the discriminative information of the network indices is preserved in the sensor space in all cases, but in the source space only for *SWi* and *λ*, as it is shown also in Table 1.

### 4.2 EEG Data Analysis

**FIGURE 9:**
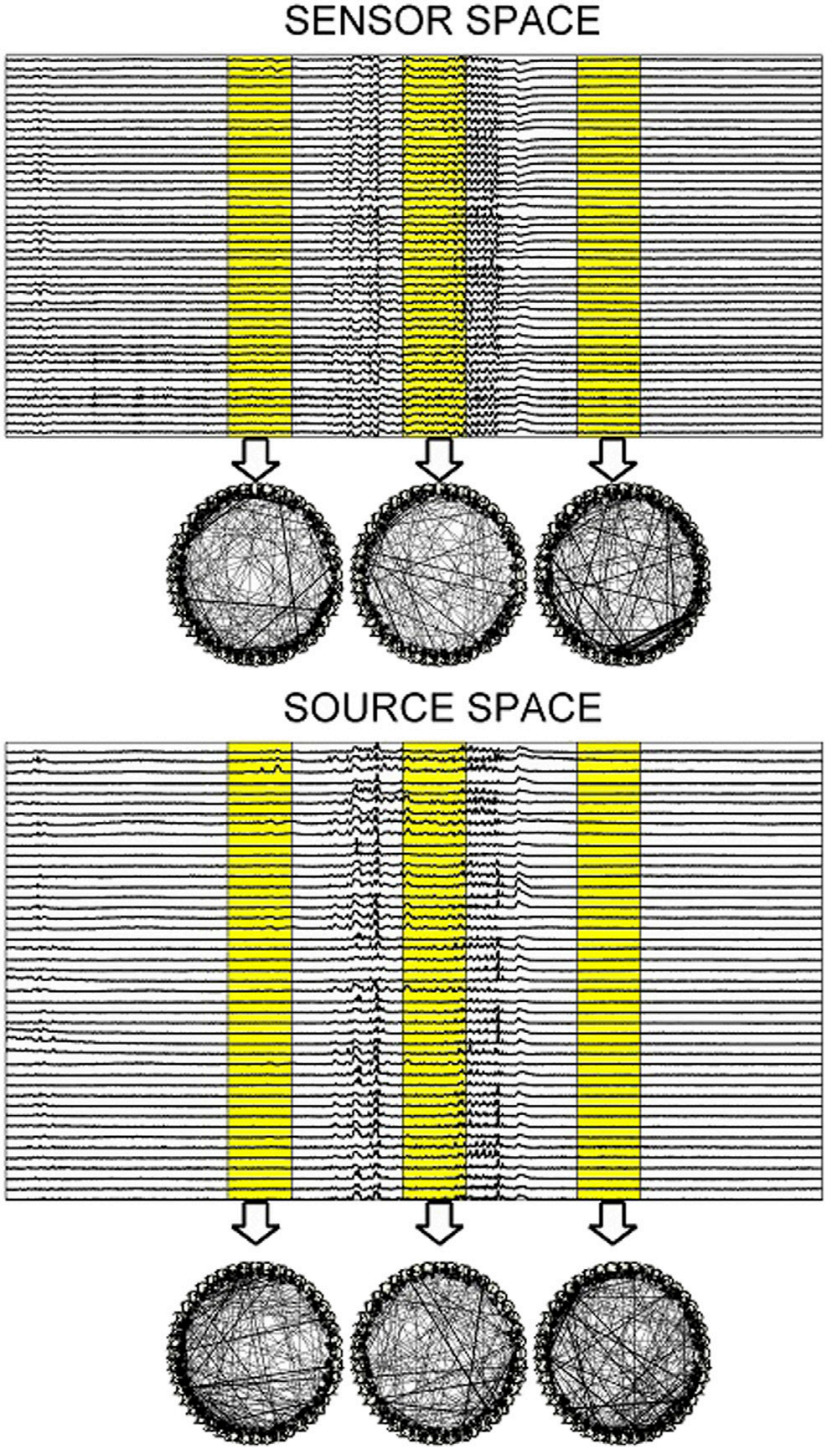
Upper panel: One epoch of S1 comprising a 10 s pre-ED period, an ED lasting 5.71 s and a 10 s post-ED period. Three time windows are highlighted (one in the pre-ED period, one during the ED and one in the post-ED period) and the respective causality networks are presented right under the arrows. Lower panel: the same but for the sLORETA transformed time series.

For the EEG data analysis, the epochs from three subjects that contain epileptiform discharges (ED) are considered. The EDs of the 7 epochs of Subject 1 (S1) have duration from 2.19 to 5.71 s and the EDs of the 9 epochs of Subject 3 (S3) from 1.94 to 5.63 s, whereas the 10th epoch is exceptionally long at 29.05 s. The 3 epochs of Subject 2 (S2) have all very short EDs of duration 1.40, 1.80, and 2.09 s. Each epoch consists of a 10 s pre-ED period, an ED period and a 10 s post-ED period. The epoch is split in sliding overlapping windows of 2 s duration and 0.5 s step on which the causality estimation is performed with PMIME. As shown in Figure 9, for each window a causality network is estimated from the initial time series and from the time series obtained after the sLORETA transformation.

**FIGURE 10:**
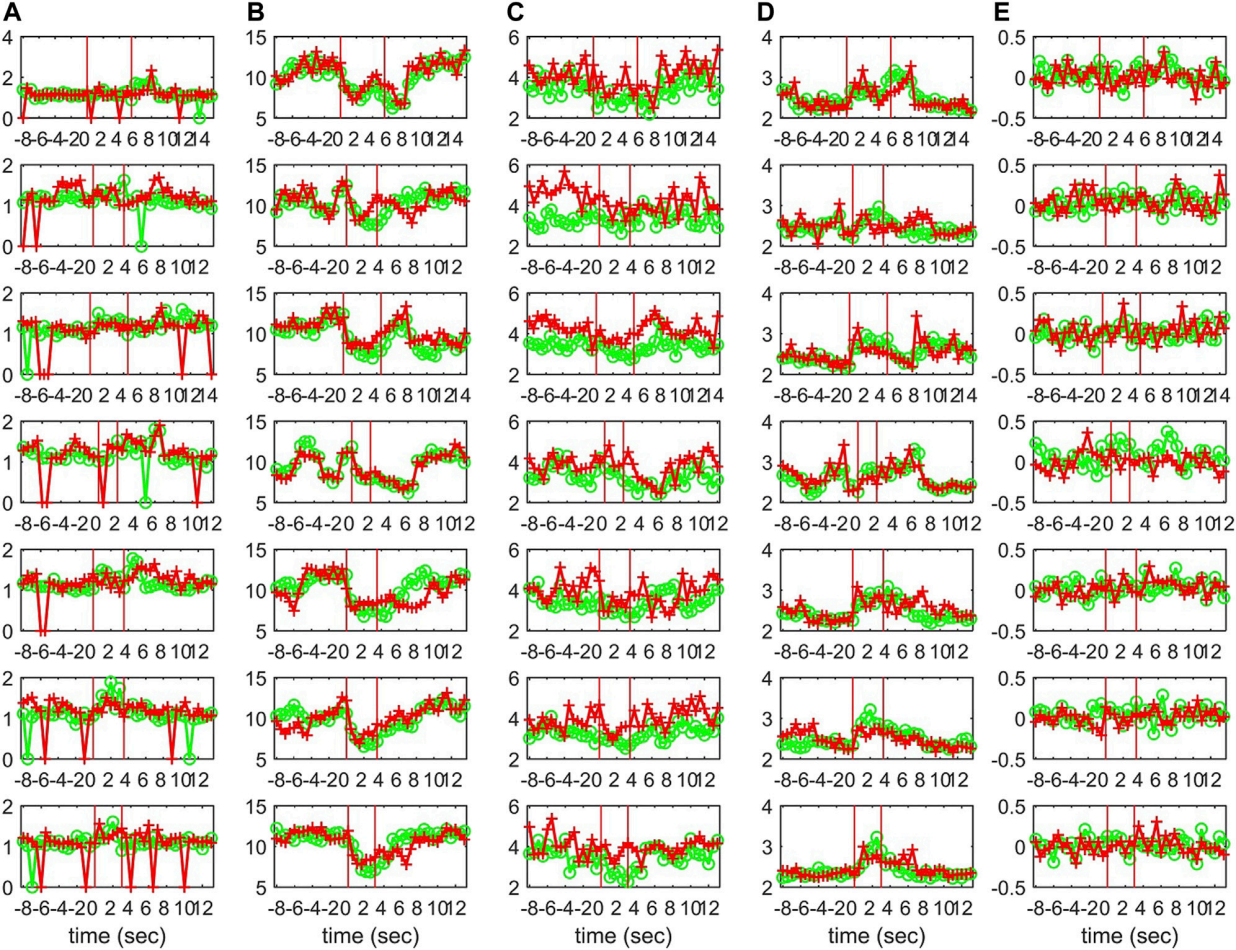
The profile of five network indices on the sensor (green line with “o” marker) and on the source space (red line with “+” marker) for 7 epochs of S1 given in the row panels: **(A)** *SWi*, **(B)** *deg* ^*m*^, **(C)** *deg* ^*SD*^, **(D)** *λ*, **(E)** *r*.

**FIGURE 11:**
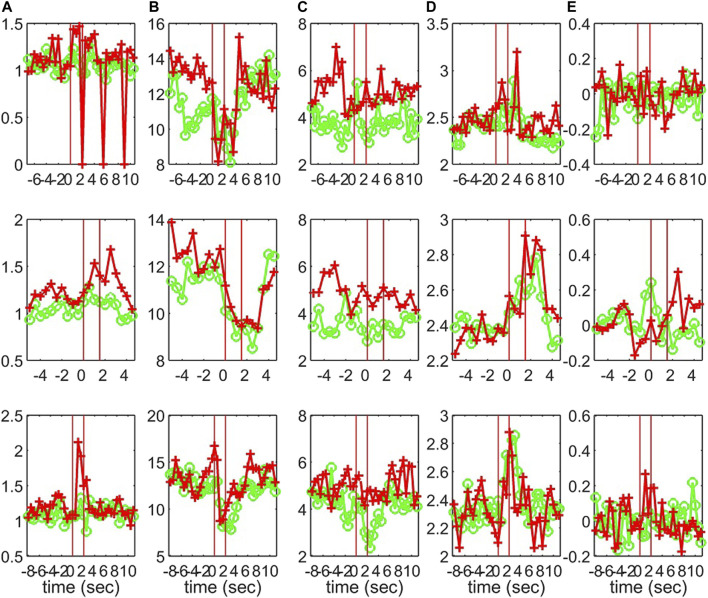
The profile of five network indices on the sensor (green line with “o” marker) and on the source space (red line with “+” marker) for 3 epochs of S2 given in the row panels: **(A)** *SWi*, **(B)** *deg* ^*m*^, **(C)** *deg* ^*SD*^, **(D)** *λ*, **(E)** *r*.

**FIGURE 12:**
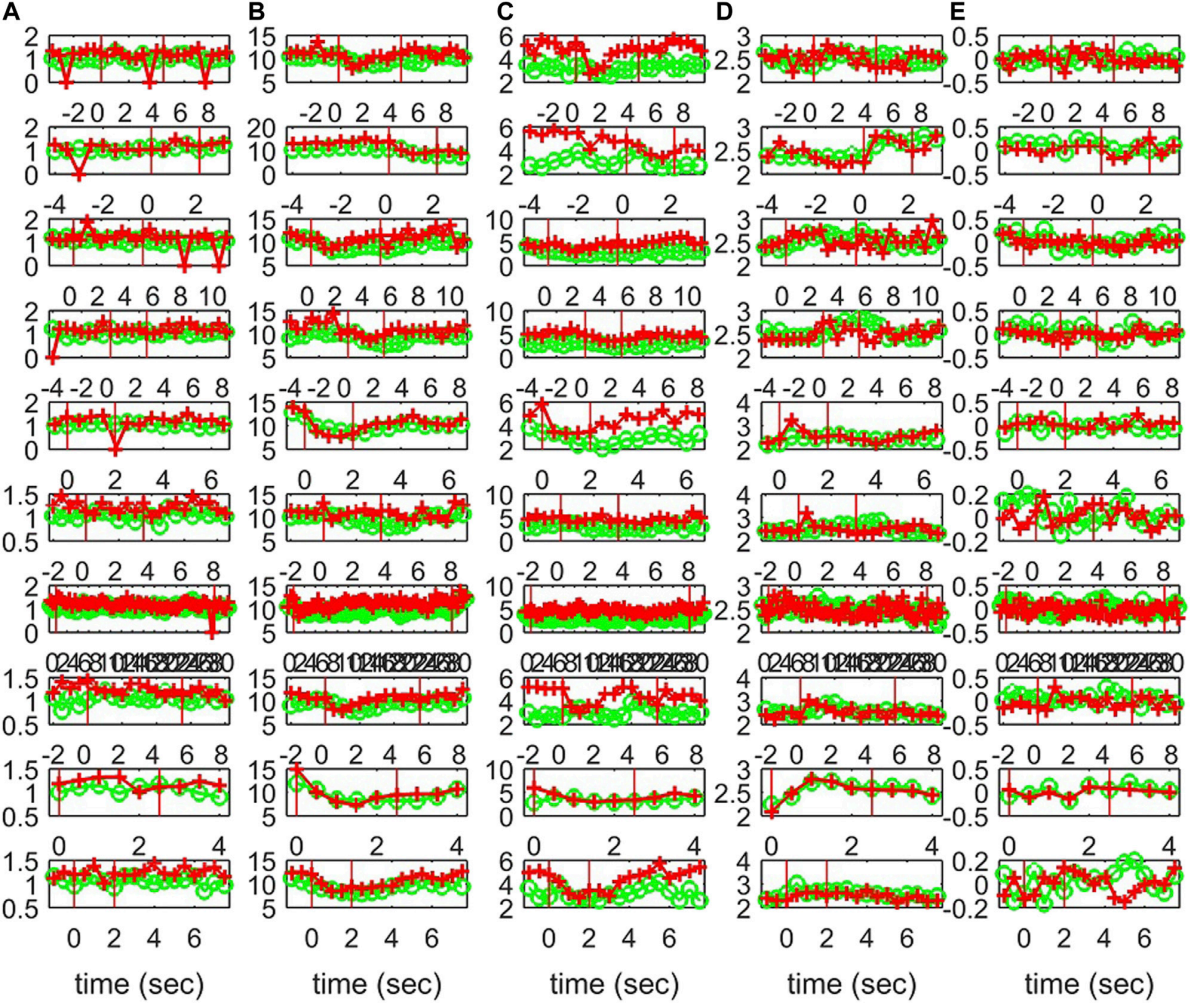
The profile of five network indices on the sensor (green line with “o” marker) and on the source space (red line with “+” marker) for 10 epochs of S3 given in the row panels: **(A)** *SWi*, **(B)** *deg* ^*m*^, **(C)** *deg* ^*SD*^, **(D)** *λ*, **(E)** *r*.

Characteristics of the causality networks are estimated by network metrics at each time window and the profiles of the metrics are shown in Figure 10 for S1, Figure 11 for S2, and Figure 12 for S3. It is observed that the network indices *deg* ^*m*^ and *λ* in both the sensor and the source space have similar estimated values during the epochs, with subtle differences at various times, e.g., for S1 the coefficient of determination *R* ^2^ is 0.52 and 0.28, respectively. In contrast, *SWi* shows significant differences in the source space at many time points, e.g., for S1 *R* ^2^ = 0.02, which is in agreement with the simulation study.

The interest is mainly in the pre-ED and ED periods where the brain state changes abruptly at the ED onset, whereas from ED to post-ED the change is gradual as the brain state recovers from the ED. To quantify the differences in the three states, before the ED (pre-ED), within the ED (ED) and after the ED (post-ED), we computed the AUROC for pre-ED vs. ED, pre-ED vs. post-ED and ED vs. post-ED in both spaces (sensor and source) and all network indices. The results for S1 and S3 are shown in Table 2, while for S2 statistical comparison is not possible as the EDs in the three episodes are very short.

**TABLE 2:** Average AUROC values over the 7 epochs of S1 and the 10 epochs of S3 for three discrimination tasks (pre-ED vs. ED, pre-ED vs. post-ED, ED vs. post-ED), both spaces (sensor, source) and five network metrics (labels in the first row).

	*SWi*	*deg* ^*m*^	*deg* ^*SD*^	*λ*	*r*
7 epochs of S1					
sensor space					
pre-ED vs. ED	0.74	0.88	0.78	0.85	0.65
pre-ED vs. post-ED	0.65	0.61	0.58	0.62	0.57
ED vs. post-ED	0.68	0.77	0.79	0.76	0.62
source space					
pre-ED vs. ED	0.59	0.80	0.77	0.75	0.60
pre-ED vs. post-ED	0.69	0.67	0.79	0.64	0.57
ED vs. post-ED	0.60	0.70	0.69	0.67	0.58
10 epochs of S3					
sensor space					
pre-ED vs. ED	0.73	0.88	0.74	0.84	0.75
pre-ED vs. post-ED	0.71	0.78	0.73	0.77	0.80
ED vs. post-ED	0.62	0.71	0.66	0.74	0.65
source space					
pre-ED vs. ED	0.68	0.84	0.79	0.78	0.67
pre-ED vs. post-ED	0.87	0.80	0.99	0.71	0.83
ED vs. post-ED	0.62	0.74	0.72	0.64	0.60

The network index *deg* ^*m*^ discriminates well the pre-ED from ED period in all episodes of the two subjects with average AUROC value 0.88 for the sensor space for both S1 and S3 and 0.80 for S1 and 0.84 for S3 for the source space. In the post-ED period, *deg* ^*m*^ recovers its initial level gradually.

For the network index *λ* the respective AUROC values are 0.85 and 0.84 for S1 and S3 for the sensor space while for the source space the corresponding AUROC are 0.75 and 0.78, respectively. The other two indices, *SW* and *r*, have low discrimination ability, but still the AUROC values are higher for the sensor space than for the source space for both subjects S1 and S3. These findings are also confirmed by the independent sample *t*-test for equal means of pre-ED and ED, as shown in Table 3, where for more episodes at the sensor space than at the source space the discrimination is established for the stringent significance level of 0.01.

**TABLE 3:** Results of the independent sample *t*-test for equal means of pre-ED and ED for subjects S1 and S3 and the five network indices as given in the first row. The numbers correspond to the ED episodes of S1 and S3 for which the *p*-value of the test is < 0.01 (for each subject first row is for the sensor space and second row for the source space).

		*SWi*	*deg* ^*m*^	*deg* ^*SD*^	*λ*	*r*
S1	sensor	2 6 7	1 3 5 6 7	1 3 6 7	1 3 5 6 7	4
	source		1 3 5 7	1 2 3 5	3 5 7	
S2	sensor	—	1 3 7 10	3	2 3 4	—
	source		2 4	2 4	4	

The results show that the network characteristics do change after the transformation to the source space at a varying degree, so that the discrimination of the pre-ED vs. ED period is less significant. This result is not in agreement to a recent report of qualitatively similar results of connectivity structure in the source activity from reconstructed scalp EEG data and the connectivity from corresponding electrocorticographic sources in primates (*Macaca mulatta*) (Papadopoulou et al., 2019).

## 5 Discussion

In this work, the level of preservation of the main network structure when estimated on the original data, also called the sensor space, and data transformed to the so-called source space has been investigated. The transformation is performed using the software sLORETA and the causality effects among the system variables are estimated with a linear method (RCGCI) and a nonlinear information based method (PMIME). After the causality network estimation, certain network characteristics are estimated by network indices in order to compare the respective network topologies in the two spaces (sensor and source).

We performed a simulation study first in order to compare the results to the predefined ground truth, defined in the sensor space. Admittedly, the true brain system is in source space and physiologically it would be more appropriate to define the simulation systems in the source space as done in (Brunner et al., 2016; Anzolin et al., 2019; Van de Steen et al., 2019). In contrast to these studies, where causal relationships of distinct sources are investigated, our study is on the overall structure of causality networks, requiring large scale systems that could only be realized under simplifications in the source space, e.g,. in (Silva Pereira et al., 2017) this was attempted assuming the same activity in all vertices (voxels) in a ROI on the cortical mesh. On the other hand, it is common to assume the simulation systems at the domain of the acquired data (here the scalp EEG). Thus, the simulation systems refer to the observed dynamics, and the focus of the study is on the change of the overall network structure of the high-dimensional coupled system under the inverse transform defined by sLORETA.

Three different network types were considered as initial coupling structures for three high-dimensional coupled dynamical systems: *K* = 20 coupled Mackey-Glass systems (CMG), *K* = 20 Hénon coupled maps (CHM) and the linear vector autoregressive process of order one on *K* = 20 variables (VAR). The objective was first to designate the differences in the topology of the causality networks in the two spaces, and then to assess whether the information the network metrics hold regarding the discrimination of the three network types was preserved after the transformation to the source space. Then we proceeded to EEG data analysis with illustrative cases from recordings of three epileptic patients that contain epileptiform discharges (EDs). The objective was again to compare the differences in the topology of the estimated causality networks in the sensor and in the source space. In addition, we tried to clarify the degree to which the topology of the networks held discriminative information regarding the pre-ED period vs. the ED and in which space the discrimination was more clear.

In the simulation study, first we showed with an analytical example that a linear transformation of the system variables can change considerably the coupling structure of the dynamical system, but it can preserve some characteristics e.g. the eigenvalues of its adjacency matrix (similar derivation was obtained in Van de Steen et al., 2019). Then, the simulation results showed that the estimated causality networks have, as expected, considerable differences in terms of the topology characteristics. Finally, the ability of certain characteristics to discriminate the initial coupling structures is reduced at a varying degree after the transformation to the source space. We note here that causality estimation inaccuracy (false positives or negatives) exists also in the sensor space (the domain of true dynamics) due to many sources, such as the time series length, the size in conjunction with the density of the true coupling network, and the type of inherent dynamics and causal relationships (the latter may encounter the common source problem related to volume conduction for EEG). However, the utilized measures RCGCI and PMIME were found to perform well in high-dimensional coupled systems and therefore they were used here to allow for a better assessment of the differences in the estimated networks in sensor and source space.

In the EEG data analysis, we showed that the characteristics of the causality network topology were altered at varying degree. Here, the small-worldness index was found to change most under the transform to source space and had a very different profile at overlapping windows across the epochs that contain ED. The average degree of the causality networks was the most discriminative characteristic regarding the pre-ED vs. ED task. Also the characteristic path length showed good discrimination ability for the same task. For both indices the ability to discriminate the two periods was better in the sensor space than in the source space. The clinical EEG data are essentially a case study providing complementary evidence that clearly corroborates the basic conclusion of the simulation analysis. Further studies, on the basis of our preliminary findings, are warranted including a larger number of subjects.

We have tactically left out of the design of the connectivity analysis the issue of field spread and volume conduction. These factors may affect the results of connectivity analysis in the sensor space and subsequently the source space. It is not known as to what extent the volume conduction affects through the inverse transform the connectivity analysis in the source space (Schoffelen and Gross, 2009), but there is reported evidence from simulation studies that it does (Anzolin et al., 2019). Functional connectivity measures suggested in the literature to account for volume conduction, such as the imaginary coherence (Nolte et al., 2004), have been considered when comparing connectivity in sensor and source space (Schoffelen and Gross, 2009; Lai et al., 2018; Demuru et al., 2020). However, these measures are bivariate (allow for indirect connections) and thus not suitable to estimate the network structure, which is the main objective of this work. The utilized causality measures here are multivariate and when estimating a causal effect they account for the presence of all other observed significant sources, including to some extent the common source due to volume conduction. Certainly, they do not explicitly account for zero-lag effects brought about by volume conduction. One of the two measures used in the study has been recently modified to account for zero-lag effects, called PMIME0 (Koutlis et al., 2019). We focused on measures of lag-causality here and leave extension to other causality measures including zero-lag effects to future work.

We have also refrained from discussing the different solutions for the inverse transform and chose the sLORETA as one of the most popular transforms. Certainly, the estimated connectivity network in source space depends on the utilized inverse transform, and this is also an open research topic gaining so far little attention, e.g., two tested inverse transforms were found to give distinctly different connectivity network characteristics in source space (Lai et al., 2018).

In view of the increasing usage of source analysis in diverse areas of neuroscience, the above data suggest that further studies in order to clarify the relationship between sensor- and source-derived data are warranted.

## Data Availability

The raw data supporting the conclusion of this article will be made available by the authors, without undue reservation.
